# Eclampsia Characteristics and Outcomes: A Comparison of Two Eras

**DOI:** 10.1155/2013/826045

**Published:** 2013-04-07

**Authors:** Mauro H. Schenone, Dorothy Miller, Jacques E. Samson, Giancarlo Mari

**Affiliations:** ^1^Department of Obstetrics and Gynecology, University of Tennessee Health Science Center, 853 Jefferson Avenue, Room E102, Memphis, TN 38163, USA; ^2^Eastern Virginia Medical School, 825 Faifax Avenue, Norfolk, VA 23507, USA

## Abstract

*Objective*. To describe the trends in incidence, characteristics, and outcomes of women with eclampsia. *Methods*. We reviewed and abstracted data from medical records of all women diagnosed with eclampsia in our institution from August 1998 to April 2011. In addition to overall characteristics and outcomes, the cases were stratified by onset: antenatal versus postnatal and early (<32 weeks of gestation) versus late antenatal cases (≥32 weeks of gestation). Comparisons were made using chi-square, Fisher's exact, Mann-Whitney *U*, and *t*-tests. A two-sided *P* < 0.05 was considered statistically significant. *Results*. We identified 87 eclampsia cases out of 59,388 deliveries; 62 cases were diagnosed before delivery, and 25 had a postnatal onset. Among the 62 antenatal cases, 41 were diagnosed before 32 weeks and 21 at or after 32 weeks of gestation. Antenatal cases had higher systolic (*P* = 0.03) and diastolic (*P* = 0.01) blood pressures, more abnormal dipstick-test proteinuria (*P* = 0.002), and lower platelet counts (*P* ≤ 0.001) than postnatal cases. Early eclampsia cases were complicated more often with HELLP syndrome than late eclampsia cases (*P* = 0.007). *Conclusion*. The occurrence of eclampsia has decreased over time. The earlier the onset is, the worse the outcome appears to be.

## 1. Introduction

Eclampsia is defined as the presence of new onset grand mal seizures in women with preeclampsia that cannot be attributed to other causes [1, 2]. It remains a rare, but significant, threat to pregnant women. The reported rate of eclampsia in the Western world is approximately 1/2000 to 1/3448 pregnancies [2, 3].

Significant changes in managing preeclampsia and eclampsia have been adopted during the last several decades, including greater competence in screening patients at higher risk for preeclampsia [4, 5], expectant management for severe preeclampsia [6–8], and increasing use of magnesium for seizure prophylaxis [9, 10]. Previous reports describing risk factors, incidence, and outcomes were based on data collected many years ago; therefore, in this study we aimed to describe the incidence, characteristics, and outcomes of women with eclampsia diagnosed during the last twelve years and managed in a single health care center in comparison with data published from our institution during a preceding era [2].

## 2. Methods

Using a perinatal database and an International Classification of Diseases 9 (ICD-9) code search in the hospital's medical record system, we identified cases of eclampsia. We reviewed medical records of all women diagnosed with eclampsia at the Regional Medical Center at Memphis from August 1998 to April 2011 and abstracted clinical and sociodemographic data. The diagnosis of eclampsia was made based on the best clinical judgment of the treating physician during the documented admission. Cases with a history of seizure disorders or with a clear etiology different from eclampsia were excluded. In our center the delivery of a patient is customarily not delayed to complete corticosteroid administration for fetal lung maturity. In addition, all patients with severe preeclampsia are given magnesium sulfate (six grams loading dose administered intravenously for fifteen to twenty minutes and two grams per hour as continuous infusion) for seizure prophylaxis. In patients with mild preeclampsia the decision to give magnesium sulfate for seizure prophylaxis is made based on the best clinical judgment of the treating physician.

We defined *antenatal* cases as those in which the diagnosis was made before delivery, *postnatal* eclampsia as those diagnosed after delivery, *early* eclampsia as those diagnosed before 32 weeks of gestation, and *late* eclampsia as those diagnosed at or after 32 weeks of gestation and before delivery.

We reported overall characteristics of eclampsia cases, maternal outcomes, and neonatal outcomes. We also reported characteristics and outcomes for groups stratified by eclampsia onset (antenatal versus postnatal and early versus late eclampsia). Additionally, we compared our results with those reported by Mattar and Sibai in 2000 in the same institution. Our study period is identified in the paper as the *second era*, and the study period of the work by Mattar and Sibai (1977–1998) [2] is referred to as the *first era*.

*Persistent neurologic deficit* was diagnosed by the clinical judgment of the treating physician or by the presence of neurologic deficits that lasted longer than what it would be expected for a postictal state. *Significant dipstick-test proteinuria *was defined as 1+ or 30 mg/dL or greater. Only in seven cases a 24-hour proteinuria was available.

Characteristics and maternal and neonatal outcomes are presented in absolute numbers, percentages, means, and standard deviations. In view of incomplete data in some cases, absolute numbers were paired with total number of cases in which the information about the characteristic being studied was available. Fisher's exact and chi-square tests were used when appropriate to compare categorical variables. When comparing continuous variables, the distribution was explored using stem-and-leaf and box-and-whisker plots. If the samples were normally distributed: Levene's test for equality of variance was performed, followed by an independent samples *t*-test. If the sample was not normally distributed, Mann-Whitney *U* test was used. The statistical software package IBM SPSS 19 (SPSS Inc., Chicago, IL, USA) and OpenEpi: Open Source Epidemiologic Statistics for Public Health, Version 2.3.1 were used for all data analyses. This study was approved and exempted from informed consent by the Institutional Review Board of the University of Tennessee.

## 3. Results

The incidence of eclampsia during the period August 1998–April 2011 was 87 cases out of 59,388 deliveries (15 cases per 10,000 deliveries); 62 (71.3%) cases were diagnosed during the antenatal period, whereas 25 (28.7%) had a postnatal onset. Out of the 62 cases diagnosed during the antenatal period, 41 (66%) cases were diagnosed before 32 weeks of gestation and 21 (34%) at or after 32 weeks of gestation. All cases of postnatal eclampsia delivered after 32 weeks of gestation. Sixteen cases with a history of seizure disorder or with a clear etiology different from eclampsia were excluded.

Table 1 shows the characteristics of patients with eclampsia during the period August 1998–April 2011 and compares the characteristics of cases with prenatal versus postnatal onset and those with a diagnosis of early versus late eclampsia. Of note, 33% of eclampsia cases did not have significant dipstick-test proteinuria. Only in seven cases 24-hour proteinuria quantification was available. One case was excluded because of concerns of an inadequate sample (report showed 11 mg/24 hours). One out of the remaining six cases had a proteinuria of less than 300 mg/24 hours (17%). The mean highest systolic (*P* = 0.03) and diastolic (*P* = 0.01) pressures were higher and the mean lowest platelet count during admission was lower (*P* ≤ 0.001) in cases of antenatal eclampsia when compared with cases of postnatal onset. A greater proportion of cases of antenatal eclampsia had a urinalysis of 1+ or more when compared with cases of postnatal eclampsia (*P* = 0.002). The mean lowest platelet count during admission was lower in cases of early eclampsia when compared with those with late onset (*P* = 0.006).

Table 2 summarizes maternal outcomes and compares such outcomes in cases with prenatal versus postnatal onset and those with a diagnosis of early versus late eclampsia.

Hemolysis, elevated liver enzymes, and low platelet count (HELLP) syndrome was more prevalent in cases of early eclampsia when compared with cases of late eclampsia (*P* = 0.007). Thirty-three percent of patients had recurrent seizures, and nineteen percent of patients had one or more seizures while receiving magnesium sulfate.  Table 3 shows neonatal outcomes and compares such outcomes in cases with prenatal versus postnatal onset and those with a diagnosis of early versus late eclampsia.

Maternal outcomes of the two eras in the same institution are compared in Table 4. The incidence of eclampsia during our study period (August 1998–April 2011, second era) was 87 cases out of 59,388 deliveries (15 cases per 10,000 deliveries); in contrast results reported by Mattar and Sibai in 2000 [2] for the period August 1977–July 1998 (first era) showed 399 cases out of 141,254 deliveries (28 cases per 10,000 deliveries) (*P* < 0.001). A summation of the two periods showed a total of 486 eclampsia cases out of 200,642 deliveries (24 cases per 10,000 deliveries).

## 4. Discussion

The incidence of eclampsia during the second era was nearly half of that reported for the first era at the same institution [2]. Among the outcomes studied, the most common maternal complication in eclampsia cases overall was recurrent seizures followed by persistent neurologic deficits.

Recent studies describing incidence, characteristics, and outcomes in cases complicated by eclampsia have been published in countries like Canada, The Netherlands, the United Kingdom, and Nigeria [11–14]. In the United States, similar studies were published about a decade ago [2, 3], before important changes to the management of eclampsia were implemented as a result of published data supporting the use of magnesium sulfate for seizure prophylaxis in cases of preeclampsia [15–25]. Our study provides new data about eclampsia after the above-mentioned changes; furthermore, this study is unique for comparing two eras in a single institution.

Our study documents an overall incidence of eclampsia of 15 cases per 10,000 deliveries, which represents a decrease in the incidence when compared with the first era. We expected such reduction, mainly because of a presumed increase in the use of magnesium sulfate for seizure prophylaxis in cases of preeclampsia during the second era studied, which starts around the time when important trials showed the superiority of magnesium sulfate for seizure prophylaxis in cases of preeclampsia/eclampsia when compared with placebo and with other antiepileptic medications [10, 16–25]. A global decrease in the incidence of eclampsia is also supported by studies in the United Kingdom and Canada [11, 13]. The eclampsia incidence in our center is higher than that reported by population-based studies [11–13] likely because of differences in the populations studied; such differences may include higher proportion of African American patients and patients of lower socioeconomic status that encounter significant difficulties in following adequate prenatal care, in addition to the fact that our hospital serves as a referral center for different hospitals in at least three states.

Our study showed that the mean highest diastolic and systolic blood pressures were higher in cases of antenatal eclampsia when compared with those with postnatal eclampsia; abnormal dipstick-test proteinuria was more common in cases of antenatal eclampsia when compared with postnatal eclampsia; HELLP syndrome was more common in cases of early eclampsia when compared with cases of late eclampsia; these results were similar to those reported for the first era [2] and support the hypothesis that the earlier the onset of eclampsia, the more severe the disease.

Although Apgar scores were lower and need for intubation was greater in the prenatal group compared with the postnatal group and in the early preeclampsia when compared with the late preeclampsia group, this is likely explained by prematurity rather than the severity of the disease.

Today, eclampsia continues to be a diagnostic and therapeutic challenge; 33% of patients had recurrent seizures, and 19% of patients had one or more seizures while receiving magnesium sulfate. Thirty-three percent of the cases did not have abnormal dipstick-test proteinuria on admission, and, therefore, a diagnosis of preeclampsia in such cases may not have preceded the onset of the disease. Furthermore, the proportion of primigravidas in the prenatal group was significantly higher than that of the postnatal group.

Despite the dogma of delivery as the ultimate treatment for preeclampsia/eclampsia, postnatal eclampsia remains an important proportion of eclampsia cases and continues to challenge our understanding of this disease.

The strengths of our study include its period and scope, which spanned more than one decade and included more than 80 cases. Furthermore, comparisons are made with the report by Mattar and Sibai in 2000 [2], in which results are based on cases that received medical care from 1977 to 1998 at our institution, thus extending the period evaluated to over three decades and including 486 eclampsia cases out of 200,642 deliveries in a single center.

The weaknesses of this study are those inherent to retrospective chart review studies, in which the data to be analyzed is limited to those documented in the medical record. In addition, only six cases had valid 24-hour proteinuria quantification and therefore our conclusions on proteinuria are mainly based on dipstick-test proteinuria, which is not the gold standard for such quantification. Nonstatistically significant results in our study may lack the power to detect clinically significant differences; therefore, caution must be used when interpreting our results.

In summary, the occurrence of eclampsia has decreased over time, but characteristics and outcomes seem to be largely unchanged, and, although rare, eclampsia can be associated with devastating complications and continues to pose a physiopathologic and therapeutic challenge. Finally, the earlier the onset of the disease is, the worse the outcomes are.

## Figures and Tables

**Table 1:** Characteristics of eclampsia cases.

Characteristic	Antenatal cases [*n*/t* (%) or mean ± SD]	Postnatal cases [*n*/t* (%) or mean ± SD]	*P* value	Early eclampsia [*n*/t* (%) or mean ± SD]	Late eclampsia [*n*/t* (%) or mean ± SD]	*P* value	All cases [*n*/*t* ^∗ ^ (%) or mean ± SD]
Maternal age (years)	22.35 ± 6.4	22.44 ± 6.0	0.954	22.81 ± 6.2	22.12 ± 6.5	0.691	22.38 ± 6.2
Race							
African American	54/62 (87%)	22/25 (88%)	>0.99	15/21 (71%)	39/41 (95%)	0.479	76/87 (87%)
Caucasian	6/62 (10%)	3/25 (12%)	>0.99	5/21 (24%)	1/41 (2%)	0.054	9/87 (10%)
Hispanic	2/62 (3%)	0/25 (0%)	>0.99	1/21 (5%)	1/41 (2%)	>0.99	2/87 (2%)
Primigravida	37/62 (60%)	9/25 (36%)	0.045	13/21 (62%)	24/41 (59%)	0.131	46/87 (53%)
Vaginal delivery	15/61 (25%)	20/25 (80%)	<0.001	1/21 (5%)	14/40 (35%)	0.015	35/86 (41%)
C/S not preceded by labor	30/61 (49%)	0/25 (0%)	<0.001	16/21 (76%)	14/40 (35%)	0.066	30/86 (35%)
C/S preceded by labor	16/61 (26%)	5/25 (20%)	0.753	4/21 (19%)	12/40 (30%)	0.545	21/86 (24%)
Induction of labor	21/57 (37%)	11/22 (50%)	0.316	2/19 (11%)	17/38 (45%)	0.144	32/79 (41%)
Assisted delivery	5/52 (10%)	1/24 (4%)	0.658	0/19 (0%)	5/33 (15%)	0.145	6/76 (8%)
Mean GA at delivery (weeks)	34.1 ± 5.1	38.4 ± 2	<0.001	27.9 ± 2.3	37.3 ± 2.6	<0.001	35.3 ± 4.8
Chronic hypertension	4/61 (7%)	0/25 (0%)	0.318	3/21 (14%)	1/40 (3%)	0.113	4/86 (5%)
Diabetes mellitus	1/61 (2%)	0/25 (0%)	>0.99	0/21 (0%)	1/40 (3%)	>0.99	1/86 (1%)
Underlying renal disease	0/60 (0%)	0/24 (0%)	—^†^	0/20 (0%)	0/40 (0%)	—^†^	0/84 (0%)
Highest uric acid (mg/dL)	8 ± 2.4	6.8 ± 2	0.2	8.7 ± 0.6	7.7 ± 0.4	0.204	7.6 ± 2.3
Highest systolic BP (mm Hg)	181.05 ± 23.6	168.23 ± 21.1	0.027	181 ± 25	181 ± 23	0.991	178 ± 24
Highest diastolic BP (mm Hg)	111.8 ± 16.3	101.48 ± 12.1	0.01	112 ± 17	111 ± 16	0.857	109 ± 16
Highest AST (mg/dL)	93.2 ± 294.2	35 ± 18.9	0.245	179 ± 490	48 ± 62	0.191	77 ± 250
Highest LDH (mg/dL)	453.9 ± 619.9	405.4 ± 363.2	0.165	706 ± 977	318 ± 199	0.225	440 ± 554
Lowest platelet count	198 ± 78	312 ± 109	<0.001	161 ± 85	218 ± 67	0.006	231 ± 101
Highest creatinine (mg/dL)	0.96 ± 0.29	0.89 ± 0.30	0.349	0.91 ± 0.2	0.99 ± 0.3	0.239	0.94 ± 0.3
≥1+ proteinuria	28/34 (82%)	7/18 (39%)	0.002	11/12 (92%)	17/22 (77%)	0.389	35/52 (67%)
Headache	36/56 (64%)	16/24 (67%)	0.838	12/19 (63%)	24/37 (65%)	0.9	52/80 (65%)
Visual disturbances	15/56 (27%)	5/24 (21%)	0.779	3/19 (16%)	12/37 (32%)	0.22	20/80 (25%)
RUQ or epigastric pain	4/56 (7%)	1/24 (4%)	>0.99	2/19 (11%)	2/37 (5%)	0.598	5/80 (6%)

Mean highest laboratory values refer to those obtained during hospital admission.

LDH: lactate dehydrogenase; AST: aspartate aminotransferase; SD: standard deviation; BP: blood pressure; CS: cesarean section; GA: gestational age; RUQ: right upper quadrant.

*t: total of cases in which information about the characteristic being studied is available; *n*: number of cases with the characteristic being studied.

^†^Unable to obtain.

**Table 2:** Maternal outcomes of eclampsia cases.

Outcome	Antenatal eclampsia [*n*/t* (%)]	Postnatal eclampsia [*n*/t* (%)]	*P* value	Early eclampsia [*n*/t* (%)]	Late eclampsia [*n*/t* (%)]	*P* value	All cases [*n*/t* (%)]
Maternal death	0/61 (0%)	0/25 (0%)	—^†^	0/21 (0%)	0/40 (0%)	—^†^	0/86 (0%)
Admission to ICU	5/51 (10%)	3/24 (13%)	>0.99	1/19 (5%)	4/32 (13%)	0.75	8/75 (10.7%)
Pulmonary edema	3/61 (5%)	1/24 (4%)	>0.99	2/21 (10%)	1/40 (3%)	0.54	4/85 (4.7%)
Respiratory arrest	5/59 (8%)	1/25 (4%)	0.84	1/21 (5%)	4/34 (12%)	0.82	6/84 (7.1%)
Intubation	10/61 (16%)	1/24 (4%)	0.24	2/21 (10%)	8/40 (20%)	0.5	11/85 (12.9%)
Cardiac arrest	1/61 (2%)	0/25 (0%)	>0.99	0/21 (0%)	1/40 (3%)	>0.99	1/86 (1.2%)
Coagulopathy	0/61 (0%)	1/25 (4%)	0.58	0/21 (0%)	0/40 (0%)	—^†^	1/86 (1.2%)
Cardiomyopathy	0/61 (0%)	0/25 (0%)	—^†^	0/21 (0%)	0/40 (0%)	—^†^	0/86 (0%)
Postpartum hemorrhage	2/61 (3%)	0/25 (0%)	>0.99	0/21 (0%)	2/40 (5%)	0.85	2/86 (2.3%)
Placental abruption	8/61 (13%)	0/23 (0%)	0.14	4/21 (19%)	4/40 (10%)	0.54	8/76 (9.5%)
HELLP syndrome	5/61 (8%)	0/24 (0%)	0.36	5/21 (24%)	0/40 (0%)	0.007	5/85 (5.9%)
Acute renal failure	5/61 (8%)	1/24 (4%)	0.91	1/21 (5%)	4/40 (10%)	0.87	6/85 (7.1%)
Aspiration pneumonia	2/61 (3%)	0/24 (0%)	>0.99	2/21 (10%)	0/40 (0%)	0.23	2/85 (2.4%)
Persistent neurologic deficits	11/61 (18%)	3/24 (13%)	0.79	4/21 (19%)	7/40 (18%)	>0.99	14/85 (16.5%)
Intracranial bleed	1/61 (2%)	0/24 (0%)	>0.99	1/21 (5%)	0/40 (0%)	0.69	1/85 (1.2%)
Blood transfusion	6/61 (10%)	0/24 (0%)	0.25	3/21 (14%)	3/40 (8%)	0.67	6/85 (7.1%)
Cases with recurrent seizures	18/56 (32%)	8/24 (33%)	0.917	6/19 (32%)	12/37 (32%)	0.993	26/80 (33%)
Cases that seized while on Mg	12/56 (21%)	3/24 (13%)	0.533	1/19 (5%)	11/37 (30%)	0.064	15/80 (19%)

ICU: intensive care unit; Mg: magnesium sulfate for seizure prophylaxis; HELLP: hemolysis, elevated liver enzymes, and low platelet syndrome.

*t: total of cases in which information about the characteristic being studied is available; *n*: number of cases with the characteristic being studied.

^†^Unable to obtain.

**Table 3:** Neonatal outcomes of eclampsia cases.

Outcome	Antenatal eclampsia [*n*/t* (%) or mean ± SD]	Postnatal eclampsia [*n*/t* (%) or mean ± SD]	*P* value	Early eclampsia [*n*/*t* ^∗ ^ (%) or mean ± SD]	Late eclampsia [*n*/t* (%) or mean ± SD]	*P* value	All cases [*n*/t* (%) or mean ± SD]
Need for cardiac massage	4/59 (7%)	0/22 (0%)	0.55	2/20 (10%)	2/39 (5%)	>0.99	4/81 (5%)
Need for intubation	22/59 (37%)	2/22 (9%)	0.014	14/21 (67%)	8/38 (21%)	<0.001	24/81 (30%)
Meconium aspiration	7/58 (12%)	5/21 (24%)	0.285	0/19 (0%)	7/39 (18%)	0.1	12/79 (15%)
Apgar score at 1 minute	5.2 ± 2.7	7.3 ± 2.7	<0.001	4 ± 2.5	5.8 ± 2.5	0.013	5.7 ± 2.8
Apgar score at 5 minutes	7.1 ± 2.1	8.4 ± 1.5	0.005	6.3 ± 2.1	7.6 ± 2	0.008	7.5 ± 2
Neonatal death	2/60 (3%)	0/23 (0%)	>0.99	1/21 (5%)	1/39 (3%)	>0.99	2/83 (2%)
Seizures	1/60 (2%)	0/23 (0%)	>0.99	0/21 (0%)	1/39 (3%)	>0.99	1/83 (1%)
Fetal death	1/61 (2%)	0/23 (0%)	>0.99	0/21 (0%)	1/40 (3%)	>0.99	1/84 (1%)
Birth trauma	0/61 (0%)	2/23 (9%)	0.145	0/21 (0%)	0/40 (0%)	—^†^	2/84 (2%)

SD: standard deviation.

*t: total of cases in which information about the characteristic being studied is available; *n*: number of cases with the characteristic being studied.

^†^Unable to obtain.

**Table 4:** Comparison of maternal outcomes of eclampsia cases stratified by era.

Outcome	Antenatal eclampsia			Postnatal eclampsia		
	First era (Mattar and Sibai) 1977–1998 [*n*/t* (%)]	Second era (current study) 1998–2011 [*n*/t* (%)]	*P* value	First era (Mattar and Sibai) 1977–1998 [*n*/t* (%)]	Second era (current study) 1998–2011 [*n*/t* (%)]	*P* value
Abruptio placentae	36/289 (12%)	8/61 (13%)	0.89	6/110 (6%)	0/23 (0%)	0.62
HELLP syndrome	39/289 (14%)	5/61 (8%)	0.26	4/110 (4%)	0/24 (0%)	0.9
DIC	20/289 (7%)	0/61 (0%)	0.03	6/110 (6%)	1/25 (4%)	>0.99
Pulmonary edema	14/289 (5%)	3/61 (5%)	>0.99	7/110 (6%)	1/24 (4%)	>0.99
Aspiration pneumonia	11/289 (4%)	2/61 (3%)	>0.99	2/110 (2%)	0/24 (0%)	>0.99
Cardiopulmonary arrest	10/289 (4%)	1/61 (2%)	0.8	6/110 (6%)	0/25 (0%)	0.57
Maternal death	2/289 (1%)	0/61 (0%)	>0.99	0/110 (0%)	0/25 (0%)	—^†^

DIC: disseminated intravascular coagulation; HELLP: hemolysis, elevated liver enzymes, and low platelet syndrome.

*t: total of cases in which information about the characteristic being studied is available; *n*: number of cases with the characteristic being studied.

^†^Unable to obtain.
